# Supplementary material for: MetaQTL: a package of new computational methods for the meta-analysis of QTL mapping experiments
Source: BMC Bioinformatics. 2007 Feb 8;8:49. doi: 10.1186/1471-2105-8-49 (PMC1808479; doi:10.1186/1471-2105-8-49)
Supplement: Additional File 7 — MetaQTL Package : jar file and tutorial. This Zip archive contains both the MetaQTL JAR file and the files of the tutorial. [file 1471-2105-8-49-S7.zip › org.inra.metaqtl/doc/org/metaqtl/util/MappingFunction.html]

MappingFunction


|  |  |  |
| --- | --- | --- |
| **Overview** \| **Package** \| **Class** \| **Use** \| **Tree** \| **Deprecated** \| **Index** \| **Help** | |  |
| **PREV CLASS**   **NEXT CLASS** | **FRAMES**    **NO FRAMES**     **All Classes** |  |
| SUMMARY: NESTED \| FIELD \| CONSTR \| METHOD | DETAIL: FIELD \| CONSTR \| METHOD |  |


---


## org.metaqtl.util Class MappingFunction

```
java.lang.Object
  org.metaqtl.util.MappingFunction
```

---

``` public final class MappingFunction extends java.lang.Object ```

define all cartogarphies functions

**Author:**
:   Jean-Baptiste Veyrieras

---

**Constructor Summary**

`MappingFunction()`


| **Method Summary** | |
| --- | --- |
| `static double` | `distance(double recombination, int function, int unit)`             Returns the distance from the recombination rate. |
| `static double` | `recombination(double distance, int function, int unit)`             Returns the recombination rate from the distance. |
| `static double` | `varianceDistance(double r, double rvar, int function, int unit)`             Returns the variance of the distance for a given recombination rate r, its variance and the mapping function. |

**Methods inherited from class java.lang.Object**

`clone, equals, finalize, getClass, hashCode, notify, notifyAll, toString, wait, wait, wait`

**Constructor Detail**

### MappingFunction

```
public MappingFunction()
```


**Method Detail**

### recombination

```
public static double recombination(double distance,
                                   int function,
                                   int unit)
```

:   Returns the recombination rate from the distance.
    unit :
    0 -> M
    1 -> cM

    :   **Parameters:**: `distance` -

---


### distance

```
public static double distance(double recombination,
                              int function,
                              int unit)
```

:   Returns the distance from the recombination rate.
    unit :
    0 -> M
    1 -> cM

    :   **Parameters:**: `recombination` -

---


### varianceDistance

```
public static double varianceDistance(double r,
                                      double rvar,
                                      int function,
                                      int unit)
```

:   Returns the variance of the distance for a given recombination
    rate r, its variance and the mapping function.
    unit :
    0 -> M
    1 -> cM

    :   **Parameters:**: `r` -: `rvar` -: `function` - **Returns:**


---


|  |  |  |
| --- | --- | --- |
| **Overview** \| **Package** \| **Class** \| **Use** \| **Tree** \| **Deprecated** \| **Index** \| **Help** | |  |
| **PREV CLASS**   **NEXT CLASS** | **FRAMES**    **NO FRAMES**     **All Classes** |  |
| SUMMARY: NESTED \| FIELD \| CONSTR \| METHOD | DETAIL: FIELD \| CONSTR \| METHOD |  |


---
